# Continuous long-term cytotoxicity monitoring in 3D spheroids of beetle luciferase-expressing hepatocytes by nondestructive bioluminescence measurement

**DOI:** 10.1186/s12896-017-0374-1

**Published:** 2017-06-20

**Authors:** Mayu Yasunaga, Yasuko Fujita, Rumiko Saito, Mitsuo Oshimura, Yoshihiro Nakajima

**Affiliations:** 1 Health Research Institute, National Institute of Advanced Industrial Science and Technology (AIST), Takamatsu, Kagawa 761-0395 Japan; 2 Department of Integrative Genomics, Tohoku Medical Megabank Organization, Tohoku University, Sendai, Miyagi 980-8573 Japan; 3 Chromosome Engineering Research Center, Tottori University, Yonago, Tottori 683-8503 Japan

**Keywords:** Luciferase, 3D culture, Artificial chromosome vector, Long-term monitoring, Cytotoxicity, Emerald Luc (ELuc), Hepatocytes, Bioluminescence

## Abstract

**Background:**

Three-dimensional (3D) spheroids are frequently used in toxicological study because their morphology and function closely resemble those of tissue. As these properties are maintained over a long term, repeated treatment of the spheroids with a test object is possible. Generally, in the repeated treatment test to assess cytotoxicity in the spheroids, ATP assay, colorimetric measurement using pigments or high-content imaging analysis is performed. However, continuous assessment of cytotoxicity in the same spheroids using the above assays or analysis is impossible because the spheroids must be disrupted or killed. To overcome this technical limitation, we constructed a simple monitoring system in which cytotoxicity in the spheroids can be continuously monitored by nondestructive bioluminescence measurement.

**Results:**

Mouse primary hepatocytes were isolated from transchromosomic (Tc) mice harboring a mouse artificial chromosome (MAC) vector expressing beetle luciferase Emerald Luc (ELuc) under the control of cytomegalovirus immediate early enhancer/chicken β-actin promoter/rabbit β-globin intron II (CAG) promoter, and used in 3D cultures. We confirmed that both luminescence and albumin secretion from the spheroids seeded in the 96-well format Cell-able^TM^ were maintained for approximately 1 month. Finally, we repetitively treated the luminescent 3D spheroids with representative hepatotoxicants for approximately 1 month, and continuously and nondestructively measured bioluminescence every day. We successfully obtained daily changes of the dose-response bioluminescence curves for the respective toxicants.

**Conclusions:**

In this study, we constructed a monitoring system in which cytotoxicity in the same 3D spheroids was continuously and sensitively monitored over a long term. Because this system can be easily applied to other cells, such as human primary cells or stem cells, it is expected to serve as the preferred platform for simple and cost-effective long-term monitoring of cellular events, including cytotoxicity.

**Electronic supplementary material:**

The online version of this article (doi:10.1186/s12896-017-0374-1) contains supplementary material, which is available to authorized users.

## Background

Cell-based reporter assays are widely used in molecular and cellular biology and pharmaceutical, biomedical, and toxicological research [1–3]. Of the reporter genes known to date, luciferases that emit light by oxidizing their substrates in a specific manner are frequently employed because their sensitivity and range of linear response are superior to those of other typical reporters, including β-galactosidase, chloramphenicol acetyltransferase, and fluorescent proteins [4]. Thus, luciferases are the most suitable reporter genes for the quantitative measurement of cellular events.

In a typical cell-based assay using luciferase, cells are destroyed at a particular time point, called the endpoint assay, enabling conventional and high-throughput assay. On the other hand, luciferases are also used in the noninvasive and longitudinal monitoring of such cellular events as gene expression, post-translational modification, and protein-protein interaction, in cellulo and in vivo [5, 6].

Among the possible luciferin-luciferase reactions, the beetle luciferase and D-luciferin (benzothiazole) pair is the best probe for long-term noninvasive monitoring of cellular events, because the luminescence generated by the reaction is highly quantitative and has an extremely low background, and D-luciferin is very stable in culture medium and easily permeates cells [7–9]. Moreover, as no external illumination is required for bioluminescence reactions, the possibility of phototoxicity caused by excitation light could be excluded. Therefore, the inherent properties of the beetle luciferin-luciferase reaction enable the longitudinal and quantitative monitoring of cellular events.

By utilizing these characteristic properties, the reporter assay using beetle luciferase has emerged as a reliable method for cytotoxicity evaluation in vitro [10] and in vivo [11]. To assess the toxicity of a test object toward small animals most directly, in vivo bioluminescence imaging has been employed for evaluating hepatotoxicity [12], renal toxicity [13], genotoxicity [14], and aryl hydrocarbon receptor activation [15], in which the increase of bioluminescence intensity accompanied by the transactivation of marker promoter or enhancer element is used as an index of toxicity. Although this imaging system realizes the noninvasive monitoring of the long-term effects of a toxicant on the same individual, the development of a more cost-effective and conventional in vitro method that can be used in longitudinal assays is strongly desired.

For this purpose, three-dimensional (3D) culture systems have been developed to mimic natural interactions among cells and to track the longitudinal metabolism or cytotoxicity of compounds, including drugs, pesticides, and bioactive substances using cancer cells, skin cells, and cardiac muscle cells [16, 17]. In particular, 3D culture systems using primary hepatocytes or cultured hepatoma cells are widely employed because among the major organs in the body, the liver is most susceptible to the toxicity of a compound. Various primary hepatocytes, including human, rat, and mouse hepatocytes, have been employed for longitudinal assays in 3D culture systems. 3D spheroids can be treated repeatedly with a compound, in which the compound is metabolized by phase I (modification by oxidation, reduction or hydrolysis) and phase II (conjugation) enzymes, inducing toxicity in the spheroids [16].

In general, cytotoxicity in 3D spheroids is assessed by typical assays, such as ATP measurement and colorimetric measurement using pigments, including methylthiazole tetrazolium (MTT) and neutral red. In these assay systems, however, continuous cytotoxicity assessment using the same spheroids is impossible because the cells must be disrupted or killed for the assays. Repeated use of the same spheroids is preferred as it would enable continuous cytotoxicity monitoring over a long term, thus resulting in the reduction of the amount of sample needed, cost savings, and facilitation of data interpretation.

To overcome this simple technical limitation, we developed a continuous long-term monitoring system in which bioluminescence of beetle luciferase from 3D spheroids was conventionally measured nondestructively. Using luminescent hepatocytes in which Emerald Luc (ELuc) [18], a green-emitting luciferase from Brazilian click beetle *Pyrearinus termitilluminans* [19], was expressed under the control of cytomegalovirus immediate early enhancer/chicken β-actin promoter/rabbit β-globin intron II (CAG) promoter, we carried out 3D culture and succeeded in the continuous monitoring of cytotoxicity induced by representative hepatotoxicants for approximately 1 month in the same 3D spheroids.

## Results

### Verification of reporter construction and correlation between bioluminescence intensity and cytotoxicity

In this study, we chose Emerald Luc (ELuc) [18], a green-emitting beetle luciferase from *P. termitilluminans* [19], to continuously monitor cytotoxicity in primary hepatocytes induced by repeated toxicant treatment for two reasons: (i) light output of ELuc from living cells is higher than that of firefly luciferase, the most widely used luciferase reporter gene, and thus high light output could be expected from hepatocytes; and (ii) D-luciferin, a luminescent substrate of ELuc, is highly stable in the culture medium and easily penetrates cells and tissues; these physicochemical properties are preferred for longitudinal and nondestructive bioluminescence measurement. Before conducting 3D cultures, we verified reporter construction in which ELuc exists downstream of CAG promoter, and the correlation between luminescence intensity and cytotoxicity using mouse fibroblasts (A9 cells) as model cells.

**Fig. 1:**
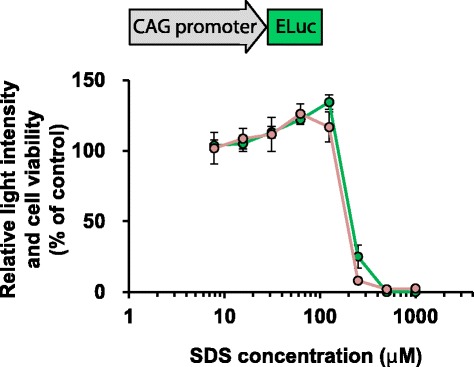
Dose-response curves of luminescence intensity and cytotoxicity in SDS-treated mouse fibroblast A9 cells. ELuc-expressing A9 cells harboring the MI-MAC vector were seeded in 96-well plates and treated with various concentrations of SDS (1000, 500, 250, 125, 63, 31, 16, and 8 μM). After incubation for 48 h in the presence of 200 μM D-luciferin, bioluminescence intensity (*green* circles) was measured nondestructively, and cell viability (*pink* circles) was assessed by WST-1 assay using the same cells. Concentration-dependent changes in bioluminescence and cell viability were expressed as percentage of untreated control (set at 100%). Error bars indicate standard deviations (*n* = 6). Schematic drawing of expression plasmid is shown in the upper panel

The A9 stable cell line was generated by the specific insertion of expression plasmid carrying the CAG promoter and ELuc, into a multi-integrase mouse artificial chromosome (MI-MAC) vector (Additional file 1: Figure S1) [20]. The resulting A9 cells have the same construction on the MI-MAC vector as transchromosomic (Tc) mice [21] used for 3D cultures, as described later. The luminescent A9 cells were seeded in 96-well plates containing culture medium to which D-luciferin and the non-selective toxicant sodium dodecyl sulfate (SDS) were added. After incubation for 48 h, first, bioluminescence intensity was nondestructively measured, and then cell viability was assessed with 2-(4-iodophenyl)-3-(4-nitrophenyl)-5-(2,4-disulfophenyl)-2*H*-tetrazolium, monosodium salt (WST-1) assay. As shown in Fig. 1, we obtained identical dose-response curves for bioluminescence and cell viability, suggesting the possibility of evaluating cytotoxicity by measuring bioluminescence from living cells, and the appropriateness of reporter construction.

### Characterization of bioluminescence properties in mouse primary hepatocytes

**Fig. 2:**
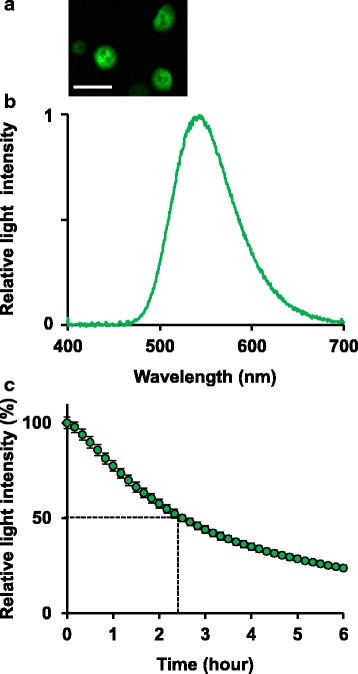
ELuc bioluminescence in mouse primary hepatocytes isolated from CAG-ELuc/MI-MAC Tc mice. Primary hepatocytes were seeded in collagen-coated 35-mm dishes and incubated for 1 day. **a** Single-cell bioluminescence imaging of ELuc-expressing hepatocytes. After 1 day of culture, the culture medium was replaced with DMEM supplemented with 10% FBS and 500 μM D-luciferin. Images were acquired using 3-min exposure time and a 4x objective lens without binning. Scale bar indicates 50 μm. **b** Emission spectrum of ELuc from the hepatocytes. After 1 day of culture, the culture medium was replaced with DMEM supplemented with 10% FBS and 200 μM D-luciferin. The spectrum was measured without destroying the cells for 1 min. **c** Stability of ELuc in the hepatocytes. After 1 day of culture, the culture medium was replaced with RM101 medium supplemented with 10% FBS, 200 μM D-luciferin, and 100 μM cycloheximide. Bioluminescence was recorded in real time for 1 min at 10-min intervals at 37 °C under 5% CO_2_ atmosphere. The maximum value was set at 100%. Error bars indicate standard deviations (*n* = 4)

Next, we characterized ELuc bioluminescence properties in mouse primary hepatocytes. Using the two-step collagenase perfusion method [22]. Primary hepatocytes were isolated from CAG-ELuc/MI-MAC Tc mice in which ELuc was expressed under the control of CAG promoter [21]. Strong bioluminescence was captured in binucleate cells by single-cell bioluminescence imaging (Fig. 2a). We nondestructively measured emission from the hepatocytes and obtained λ_max_ at 540 nm, consistent with that from ELuc-expressing living NIH3T3 cells [18, 23] (Fig. 2b). These results indicate that ELuc was adequately expressed in the mouse primary hepatocytes.

When ELuc luminescence from the hepatocytes was measured in real time in the presence of cycloheximide, a protein synthesis inhibitor, the functional half-life of ELuc in the primary hepatocytes was estimated to be approximately 2.4 h (Fig. 2c), which is appropriate for monitoring rapid cellular responses. As the half-lives of ELuc in A9 and NIH3T3 cells are 5.9 [24] and 9.8 [18] hours, respectively, ELuc is relatively unstable in the hepatocytes, probably due to the rapid degradation of ELuc mRNA and/or protein.

### Function and bioluminescence in 3D spheroids

We conducted 3D culture of primary hepatocytes isolated from CAG-ELuc/MI-MAC Tc mice in which ELuc was expressed under the control of CAG promoter. The hepatocytes were isolated by the two-step collagenase perfusion method, and viable hepatocytes were purified by Percoll centrifugation, as described in Methods. The hepatocytes were seeded in the 96-well format Cell-able^TM^, a micropatterned 3D culture system [25], in which 3T3-Swiss albino cells were seeded as feeder cells 1 day before hepatocyte seeding. The culture medium was refreshed every 2 to 3 days, unless otherwise noted, and the culture was continued for approximately 1 month.

**Fig. 3:**
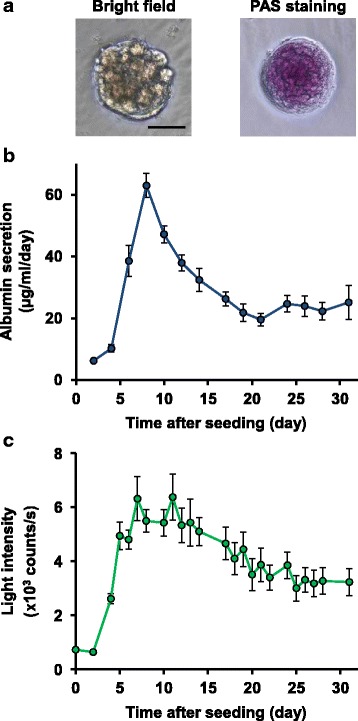
3D culture of primary hepatocytes isolated from CAG-ELuc/MI-MAC Tc mice. Primary hepatocytes were seeded in the 96-well format Cell-able^TM^, in which feeder cells (3T3-Swiss albino) were pre-seeded 1 day before hepatocyte seeding, at the density of 4 × 10^4^ cells/well. The culture medium containing 300 μM D-luciferin was refreshed every 2 to 3 days. **a** Morphology (*left panel*) and glycogen accumulation (*right pane*l) of a 3D spheroid on culture day 7. Glycogen was stained with PAS. Scale bar indicates 50 μm. **b** Sequential changes of albumin secretion from 3D spheroids. Albumin concentration in the culture medium on the indicated culture day was measured by ELISA, and divided by culture day. Error bars indicate standard deviations (*n* = 8). **c** Sequential changes of ELuc bioluminescence from 3D spheroids. Bioluminescence from the 3D spheroids shown in (**b**) was measured nondestructively for 3 sec in each well using a luminometer. Error bars indicate standard deviations (*n* = 8)

On culture day 7, spheroids were clearly observed with glycogen accumulation, which was stained by the periodic acid-Schiff’s (PAS) reaction (Fig. 3a). The expression of genes in the hepatocytes, including genes encoding phases I and II enzymes, was also confirmed by microarray analysis of the 3D spheroids on culture day 7 (Additional file 2: Table S1). To verify the expression and maintenance of hepatocyte function during the 3D culture, we measured albumin secretion in the culture medium. As shown in Fig. 3b, albumin secretion started to increase from culture day 4, and peaked on culture day 8. The albumin secretion decreased thereafter but continued until culture day 31, demonstrating that mouse primary hepatocyte function persisted for approximately 1 month in our 3D culture conditions. We found that albumin secretion was not inhibited during the culture period even in the presence of D-luciferin at 300 μM, the concentration used in subsequent experiments (Additional file 3: Figure S2), indicating that D-luciferin inhibits neither spheroid formation nor the expression of hepatocyte function.

Continuous measurement of ELuc luminescence from the same 3D spheroids as those shown in Fig. 3b revealed that the luminescence also persisted for 1 month (Fig. 3c), and the luminescence intensity was sufficient for cytotoxicity assessment by nondestructive bioluminescence measurement. Interestingly, the sequential changes of luminescence intensity during the 3D culture were similar to those of albumin secretion, suggesting that ELuc luminescence intensity may reflect hepatocyte function.

Together, we concluded that both the 3D culture system and the bioluminescence measurement system used in this study satisfy the conditions for cytotoxicity evaluation in 3D spheroids subjected to repeated treatment with a test compound.

### Continuous bioluminescence monitoring of 3D spheroids during repeated treatment with hepatotoxicants

**Fig. 4:**
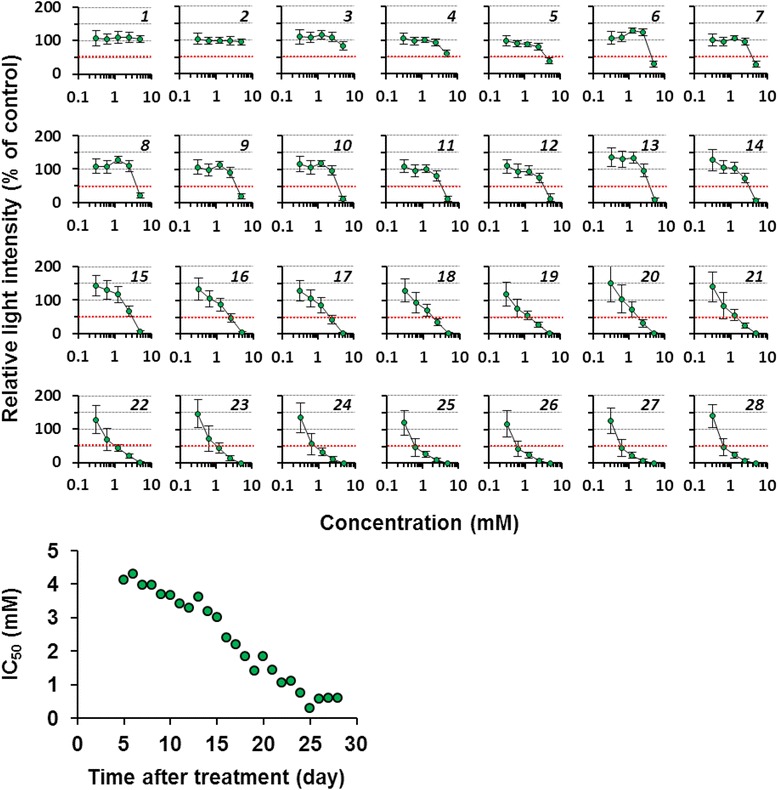
Daily changes of dose-response curves of ELuc bioluminescence from acetaminophen-treated 3D spheroids. Primary hepatocytes were seeded in the 96-well format Cell-able^TM^, in which feeder cells (3T3-Swiss albino) were pre-seeded 1 day before hepatocyte seeding, at the density of 4 × 10^4^ cells/well. The culture medium containing 300 μM D-luciferin and acetaminophen (5, 2.5, 1.3, 0.6, and 0.3 mM) was refreshed every 2 to 3 days. Bioluminescence from the spheroids on the indicated culture day was measured nondestructively every day for 5 s in each well using a luminometer. The dose-dependent change of bioluminescence intensity was expressed as the percentage of untreated control (set at 100%). Bioluminescence intensity at 50% was marked by a dotted red line. Italic digits in the figures indicate culture day after initial treatment. Sequential changes of IC_50_ values are shown in bottom panels. Error bars indicate standard deviations (*n* = 4)

**Fig. 5:**
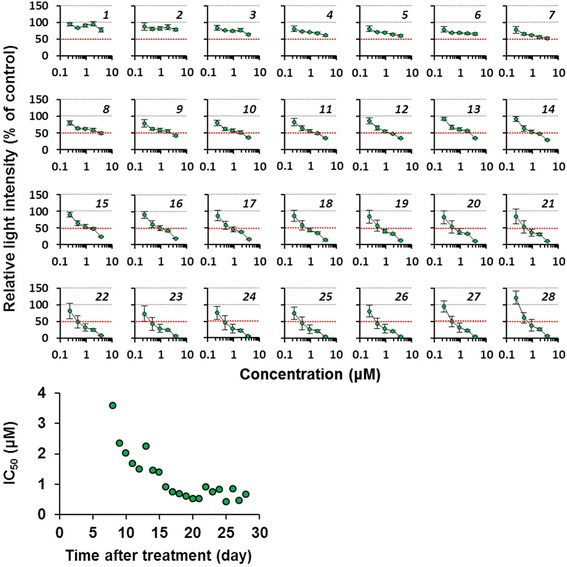
Daily changes of dose-response curves of ELuc bioluminescence from 3-methylcholanthrene-treated 3D spheroids. The culture medium containing 300 μM D-luciferin and 3-methylcholanthrene (5, 2.5, 1.3, 0.6, and 0.3 μM) was refreshed every 2 to 3 days. 3D cultures and bioluminescence measurement were performed as described in Methods and legend in Fig. 4. The dose-dependent change of bioluminescence intensity was expressed as the percentage of untreated control (set at 100%). Bioluminescence intensity at 50% was marked by a dotted red line. Italic digits in the figures indicate culture day after initial treatment. Sequential changes of IC_50_ values are shown in bottom panels. Error bars indicate standard deviations (*n* = 4).

**Fig. 6:**
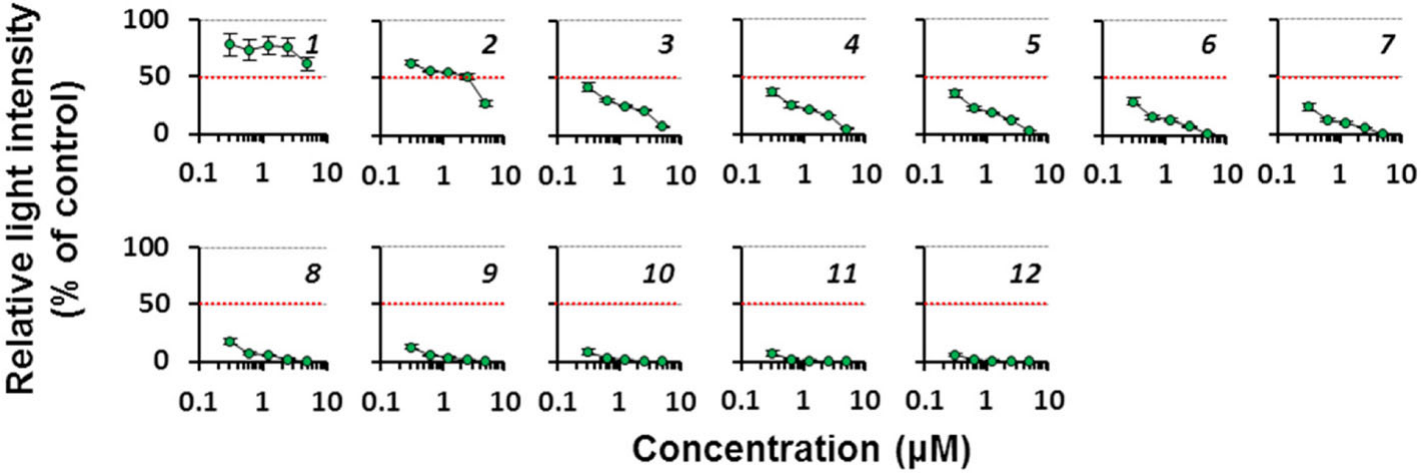
Daily changes of dose-response curves of ELuc bioluminescence from aflatoxin B1-treated 3D spheroids. The culture medium containing 300 μM D-luciferin and aflatoxin B1 (3.7, 1.9, 0.9, 0.5, and 0.2 μM) was refreshed every 2 to 3 days. 3D cultures and bioluminescence measurement were performed as described in Methods and legend in Fig. 4. The dose-dependent change of bioluminescence intensity was expressed as the percentage of untreated control (set at 100%). Bioluminescence intensity at 50% was marked by a dotted red line. Italic digits in the figures indicate culture day after initial treatment. Error bars indicate standard deviations (*n* = 4).

Finally, we verified the utility of the continuous nondestructive bioluminescence monitoring of 3D spheroids for assessing hepatocyte toxicity induced by the longitudinal treatment of representative hepatotoxicants. Mouse primary hepatocytes prepared from CAG-ELuc/MI-MAC Tc mice were seeded in the 96-well format Cell-able^TM^. After culture day 6, the 3D spheroids were treated with acetaminophen, 3-methylcholanthrene or aflatoxin B1, all of which are known to induce hepatotoxicity and hepatic dysfunction when metabolized by specific cytochromes [16]. The culture medium containing D-luciferin and a representative hepatotoxicant was refreshed every 2 to 3 days, and the 3D culture was continued for approximately 1 month. Bioluminescence in the same culture plate was measured nondestructively every day during the culture using a luminometer (Figs. 4–6 and Additional file 4: Figure S3).

As shown in Fig. 4, in the acetaminophen-treated spheroids, ELuc luminescence showed no marked changes until treatment day 3, but was gradually decreased from treatment day 4 at the highest concentration tested. After treatment day 5, ELuc luminescence was slowly decreased by repeated acetaminophen treatment, although the luminescence did not change at the lowest concentration tested. The first 50% inhibitory concentration (IC_50_) for ELuc luminescence was determined on treatment day 5, and the IC_50_ values showed a gradual decrease with increasing culture period (Fig. 4, bottom panel). Similar ELuc luminescence decrease was noted by repeated 3-methylcholanthrene treatment (Fig. 5, top panels), but the sequential decrease of IC_50_ values was slightly faster than that of the acetaminophen-treated spheroids (Fig. 5, bottom panel). In the aflatoxin B1 treated spheroids, ELuc luminescence was rapidly decreased, and the luminescence was completely suppressed on treatment day 10 at all the concentrations tested (Fig. 6). The intermediate precision of luminescence measurements with a luminometer during the experimental period was confirmed by using a standard light emitting diode (LED) plate, and the coefficient of variation (CV) was 2.1% (Additional file 5: Figure S4), demonstrating good intermediate precision of the measurements.

## Discussion

We succeeded in the continuous long-term monitoring of the daily changes of cytotoxicity induced by repeated treatment of 3D spheroids with representative hepatotoxicants by nondestructive ELuc bioluminescence measurement. To our knowledge, this work is the first to achieve the nondestructive monitoring of bioluminescence from 3D spheroid primary culture cells for approximately 1 month.

Bioluminescence measurement using beetle luciferase offers several advantages in monitoring cellular responses, including the extremely low background of the luciferin-luciferase reaction that results in a high signal-to-noise ratio, and the very wide dynamic range (up to seven or eight orders of magnitude) when a photomultiplier tube is used as the detector. These advantages result in the sensitive detection of bioluminescence from 3D spheroids.

In general, in vitro cytotoxicity is assessed by typical assays, including ATP assay using beetle luciferase protein and colorimetric assays using pigments, such as MTT, WST or neutral red. Regardless of the assay systems, however, the cells must be disrupted or killed at each time point. Thus, many cells and culture plates are needed when time-course measurements are performed. In contrast, nondestructive bioluminescence measurement can continuously assess cytotoxicity at an arbitrary time point using the same cells.

In 3D cultures, feeder cells are often used, which serve to maintain the morphology and function of 3D spheroids [16]. Indeed, in our 3D culture system, when feeder cells (3T3-Swiss albino) were used, both bioluminescence intensity and albumin secretion were high and stably maintained compared to those of the feeder-free 3D culture (data not shown). Thus, feeder cells play an important role in 3D culture. However, it is impossible to discriminate cytotoxicity to 3D spheroids and feeder cells when cytotoxicity is assessed by typical assays, such as ATP, MTT, and neutral red uptake assays, which may lead to misinterpretation of the results. The lactate dehydrogenase (LDH) assay that measures the activity of LDH released from damaged cells, or the fluorescence or absorbance measurement of the membrane-permeable dye, resazurin, which emits fluorescence or changes its color upon reduction in viable cells, is a candidate for continuous cytotoxicity monitoring as cell disruption is not required. However, it is also impossible to discriminate LDH activity or the fluorescence or absorbance of resazurin between 3D cells and feeder cells. In this regard, in the bioluminescence measurement conducted in this study, the effect of toxicant on 3D spheroids can be assessed accurately because luminescence is emitted by only 3D spheroids.

Co-culture systems, in which hepatocytes and non-parenchymal cells, including Kupffer cells, stellate cells, or vascular endothelial cells, are co-cultured in a 3D environment, have been developed, and these have contributed to the improvement of hepatocyte function and the high-sensitivity detection of indirect hepatotoxicity via Kupffer cell activation [16, 17]. As in the case of feeder cells, however, it is quite difficult to discriminate toxicity toward hepatocytes and non-parenchymal cells. We assume therefore that the nondestructive bioluminescence measurement method would be also useful for co-culture systems for the direct monitoring of toxicity toward hepatocytes. Moreover, as regards the advantage of bioluminescence measurement, the linear dynamic range in bioluminescence measurement is generally superior to that in fluorescence or absorbance measurements when a photomultiplier tube is used as the detector.

Although the bioluminescence measurement shows potential for the continuous monitoring of cytotoxicity in 3D cultures, in some cases, we may need to pay careful attention because some compounds may inhibit the luciferin-luciferase reaction [26, 27] or stabilize luciferase protein in cells by interacting with it [28], even though the toxicants used in this study do not affect D-luciferin and ELuc reaction (data not shown). This issue may be resolved by the combined use of multiple luciferases [6], such as the dual luciferase reporter assay that harnesses firefly and *Renilla* luciferases by utilizing substrate specificity, or a multicolor luciferase assay that uses multiple luciferases that produce different color emissions by reacting with D-luciferin.

In the present study, the repeated dose test was conducted using luminescent 3D spheroids. As shown in Figs. 4–6, acetaminophen, 3-methylcholanthrene, and aflatoxin B1 induced sequential decreases of ELuc bioluminescence. As the bioluminescence decrease agreed with cell viability decrease as confirmed by parallel measurements of bioluminescence and cell viability by the WST-1 assay in ELuc-expressing A9 cells (Fig. 1), it is reasonable to consider that the application of these compounds to 3D spheroids induces dose- and time-dependent cytotoxicity in the 3D spheroids.

In addition, we noted that bioluminescence intensity did not change markedly in 2D cultures in which hepatocytes prepared from CAG-ELuc/MI-MAC Tc mice were cultured in collagen-coated 96-well plates for 3 days in the presence of acetaminophen, 3-methylcholanthrene, and aflatoxin B1, even when the same hepatotoxicant concentrations as those used in the 3D cultures were applied (data not shown). This may reflect an insufficient exposure period and/or cytochrome expression in the 2D culture system, suggesting the applicability of the 3D spheroids and the 3D culture system used in this study.

In the 3D culture, ELuc bioluminescence persisted for approximately 1 month, during which it showed a rapid increase after culture day 2 and peaked around culture days 7 to 11 (Fig. 3c). This rapid luminescence increase might be due to recovery from damage by Percoll treatment and/or enhancement of hepatocyte function accompanying the formation of spheroids, because the time-dependent change of bioluminescence from the hepatocytes was similar to that of albumin secretion (Fig. 3b). In addition, it has been reported that an organic anion transporter is involved in the incorporation of D-luciferin, the substrate for ELuc, into the cells [29]. In the 3D culture, we were able to confirm the expression of many organic anion transporters on culture day 7 by microarray analysis (Additional file 2: Table. S1). Thus, the rapid increase and maintenance of ELuc bioluminescence in the 3D culture may also reflect the increased organic anion transporter expression associated with the enhancement of hepatocyte function.

## Conclusions

In this study, we developed a simple and cost-effective cytotoxicity assay system that enabled us to continuously assess cytotoxicity in the same 3D spheroids. This monitoring system is based on bioluminescence, an artificial chromosome vector, and 3D culture techniques. As the artificial chromosome vector, including the MI-MAC vector used in this study, can be transferred from host cells, such as CHO or A9 cells, to recipient cells by the microcell-mediated chromosome transfer method [30], the bioluminescence monitoring system developed in this study would be applicable to other 3D cultures that use human primary hepatocytes, such as HepaRG cells, solving the problem of the difference in metabolism between mouse and human. In addition, cancer cells or differentiated cells from induced pluripotent stem cells would be also applicable. Moreover, a longitudinal 3D culture assay system would be developed, which would examine the molecular mechanisms underlying cytotoxicity when an additional luciferase, such as red-emitting beetle luciferase or secreted luciferase, is introduced into the system developed in this study, in which CAG-ELuc is used as the internal control reporter and another luciferase is used as the test reporter to monitor signaling pathways or marker gene expression.

## Methods

### Cell culture

Mouse fibroblast A9 cells harboring the MI-MAC vector (hereinafter referred to as A9-MI-MAC) [20] and 3T3-Swiss albino cells (Japanese Collection of Research Bioresources Cell Bank, Osaka, Japan) were grown in Dulbecco’s Modified Eagle’s Medium (DMEM, Sigma-Aldrich, St. Louis, MO) supplemented with 10% fetal bovine serum (FBS, ICN Biochemicals, Aurora, OH) in a humidified atmosphere containing 5% CO_2_ at 37 °C.

### Generation of stable cell lines

To construct a reporter plasmid carrying ELuc, ELuc cDNA was excised with *Hin*dIII and *Not*I from pELuc-test (Toyobo, Osaka, Japan), and the fragment was ligated into the HindIII/NotI sites of pCAG-GLuc (a gift from Dr. Ohbayashi), in which the attL1 and attL2 sites are flanked by upstream and downstream of CAG promoter and simian virus (SV) 40 polyA signal, respectively. The expression cassettes containing CAG promoter, ELuc, and SV40 poly A signal were recombined into pNeo-φC31 attB of pφC31-Neo vector [31] by the LR reaction using LR Clonase II Enzyme Mix (Invitrogen, Durham, NC), yielding pφC31-Neo-CAG-ELuc. To make a stable cell line, A9-MI-MAC were seeded in six-well plates at the density of 6 × 10^5^ cells per well 1 day before transfection. Three micrograms of pφC31-Neo-CAG-ELuc was co-transfected with 1 μg of φC31 recombinase expression plasmid pCMV-φC31 [31] using Lipofectamine PLUS (Invitrogen) according to the manufacturer’s instructions. The transfected cells were seeded in 10-cm dishes 1 day after transfection, and subcultured for selection with 800 μg/ml G418 (Nacalai Tesque, Kyoto, Japan). Integration of transgene into the corresponding site on the MI-MAC vector was confirmed by genomic PCR.

### Bioluminescence measurement and cytotoxicity assay using A9 stable cells

The A9 stable cell line in which ELuc was expressed under the control of CAG promoter was seeded in a 96-well clear bottom plate (Nunc, Wiesbaden, Germany) at the density of 1 × 10^4^ cells per well. After 1 day, the medium was replaced with DMEM without phenol red (Gibco-BRL, Grand Island, NY) but supplemented with 10% FBS, 25 mM Hepes/NaOH (pH 7.0, Sigma-Aldrich), and 200 μM D-luciferin (Toyobo), and incubated for 48 h in the presence of various concentrations of SDS (Nacalai Tesque). Bioluminescence was nondestructively measured for 5 s in each well at 37 °C using a microplate-type luminometer (AB-2350 Phelios, ATTO, Tokyo, Japan), and then viability of the same cells was measured using the cell proliferation reagent WST-1 (Takara, Shiga, Japan) according to the manufacturer’s protocol. Absorption was measured at 440 nm using a microplate reader Infinite F2000 PRO (Tecan, Männedorf, Switzerland).

### Animals

To isolate primary hepatocytes, we used 8- to 13-week-old male CAG-ELuc/MI-MAC Tc mice [21]. The Tc mice were backcrossed to ICR mice (SLC, Shizuoka, Japan) for more than three generations. The animal experimental protocols were approved by the Institutional Animal Care and Use Committee of the National Institute of Advanced Industrial Science and Technology. All animal experiments were carried out in accordance with the approved protocols.

### Isolation of primary hepatocytes

Primary hepatocytes were isolated by a two-step collagenase perfusion method according to a previous report [22] with modifications. After CAG-ELuc/MI-MAC Tc mouse was anesthetized with 3% isoflurane gas using small animal anesthetizer (MK-AT200, Muromachi Kikai, Tokyo, Japan), the liver was perfused with liver perfusion medium (Gibco-BRL) via the hepatic portal vein and immediately the abdominal inferior vena cava was cut to induce bleeding. After the liver had been freed of blood, liver perfusion medium (Gibco-BRL) was replaced with 0.5 mg/ml collagenase buffer (Sigma-Aldrich) for 4 min. The perfusion rate of 5 ml/min and the temperature of around 40 °C were maintained for both perfusates and collagenase buffer during the entire procedure. After perfusion, the liver was rapidly transferred to a sterile petri dish and shredded in cold hepatocyte wash medium (Gibco-BRL). The medium containing shredded liver was filtered through a 100 μm cell strainer (Becton Dickinson, Bedford, MA). After washing by low-speed centrifugation at 50 g for 3 min at 4 °C, the hepatocytes were suspended in the appropriate medium for subsequent experiments.

### Single cell bioluminescence imaging

For bioluminescence imaging, isolated hepatocytes were seeded in a 35-mm dish coated with Type I-A collagen (Nitta Gelatin, Osaka, Japan) and incubated for 1 day. The culture medium was replaced with DMEM without phenol red (Gibco-BRL) but supplemented with 10% FBS, 25 mM Hepes/NaOH (pH 7.0, Sigma-Aldrich), and 500 μM D-luciferin potassium salt (Toyobo). Bioluminescence imaging was performed using a luminescence microscope (CellGraph, ATTO) at 37 °C. Charged-coupled device images were acquired using a 4× objective lens (NA, 0.9; Nikon, Tokyo, Japan) at 1 × 1 binning of the 512 × 512 pixel array.

### Spectral measurement

Isolated hepatocytes were seeded in 35-mm dishes coated with Type I-A collagen (Nitta Gelatin) at the density of 8 × 10^5^ cells per dish and incubated for 1 day. The medium was replaced with DMEM without phenol red (Gibco-BRL, Grand Island, NY) but supplemented with 10% FBS, 25 mM Hepes/NaOH (pH 7.0, Sigma-Aldrich), and 200 μM D-luciferin potassium salt (Toyobo). The dish was placed on the sample stage of a spectrophotometer (AB-1850, ATTO) and spectral measurements were carried out without destroying cells for 1 min with a slit width of 1 mm.

### Stability of ELuc in primary hepatocytes

Isolated hepatocytes were seeded in 35-mm dishes coated with Type I-A collagen (Nitta Gelatin) at the density of 8 × 10^5^ cells per dish 1 day before measurement. The culture medium was replaced with RM101 medium (Toyo Gosei, Tokyo, Japan) supplemented with 25 mM Hepes/NaOH (pH 7.0; Sigma-Aldrich), 200 μM D-luciferin potassium salt (Toyobo), and 100 μM cycloheximide (Nacalai Tesque), and overlaid with 1.5 ml of mineral oil (Sigma-Aldrich). Bioluminescence was recorded in real time for 1 min at 10-min intervals using a dish-type luminometer (AB2500 Kronos, ATTO) in a humidified atmosphere containing 5% CO_2_ at 37 °C.

### 3D cultures

For 3D cultures, viable hepatocytes were purified from the isolated hepatocyte fraction described above by iso-density Percoll centrifugation in 45% Percoll (Sigma-Aldrich) at 50 g for 1 min at 4 °C, and the hepatocytes were suspended in RM101 medium (Toyo Gosei). Viability of the hepatocytes was assessed by trypan blue exclusion, and suspensions with viability of over 90% were used.

Feeder cells (3T3-Swiss albino) were seeded at the density of 2 × 10^4^ cells per well on the 96-well format Cell-able^TM^ (Toyo Gosei), and incubated for 1 day. The purified hepatocytes were seeded at the density of 4 × 10^4^ cells per well in the presence of D-luciferin unless otherwise noted. The culture medium was refreshed every 2 or 3 days. Exchanged culture medium was stocked at −30°C until use for secreted albumin measurement.

### Periodic acid-Schiff’s staining

To confirm glycogen accumulation in the 3D spheroids, after culture day 7, 3D spheroids were stained with PAS using a PAS staining kit (Muto Pure Chemicals, Tokyo, Japan) according to the manufacturer’s protocol. Microscopy images were acquired with a microscope using a 20× objective lens (BZ-X710, Keyence, Osaka, Japan).

### Measurement of secreted albumin

After thawing the stocked medium, albumin concentration was measured using the Mouse Albumin ELISA Quantitation Set (Bethyl Laboratories, Montgomery, TX) according to the manufacturer’s protocol. The absorbance was measured at 450 nm using a microplate reader (Bio-Rad, Hercules, CA).

### Repeated treatment of 3D spheroids with representative hepatotoxicants

Acetaminophen, 3-methylcholanthrene, and aflatoxin B1 were purchased from Sigma-Aldrich. The compounds were dissolved in dimethyl sulfoxide (DMSO, Sigma-Aldrich) and diluted with culture medium. This preparation was carried out immediately before use every time. The final concentration of DMSO was less than 0.1%. After culture day 6, 3D spheroids were exposed to each compound, and culture medium containing 300 μM D-luciferin and compound was refreshed every 2 or 3 days. Bioluminescence from the 3D spheroids was measured nondestructively for 5 s in each well using Phelios (ATTO). Reproducibility of the luminescence measurement during the experimental period was confirmed with the standard LED plate TRIANT (WSL-0001, ATTO).

### DNA microarray

Total RNA was isolated from feeder cells or 3D spheroids on culture day 7 using an RNeasy Mini Kit (Qiagen, Valencia, CA) according to the manufacturer’s instructions. Total RNA was amplified and labeled with cyanine-3 (Cy-3) using a Low Input Quick Amp Labeling Kit (Agilent Technologies, Palo Alto, CA) according to the manufacturer’s protocol. Cy3-labeled cRNA was hybridized to Mouse Whole Genome Oligo DNA Microarray ver. 2.0 (Agilent Technologies) containing 44,397 features representing 39,429 biological probes using a Gene Expression Hybridization Kit (Agilent Technologies) at 65 °C for 17 h, and scanned with a SureScan Microarray Scanner (Agilent Technologies). Signals in the scanning image were converted into intensity values using Feature Extraction version 11.5.1.1 (Agilent Technologies). Expression raw data from all arrays were analyzed using GeneSpring GX 13.0 software (Agilent Technologies), and global normalization was performed. After the normalization, extremely low intensity probes were excluded, leaving 39,365 probes for analysis. Genes specifically expressed in the 3D spheroids were identified by applying a cutoff of ±1.5-fold and a paired *t*-test with *p* < 0.05.

## Additional files

Additional file 1: Figure S1. Schematic diagram of site-specific insertion of reporter plasmid carrying ELuc into MI-MAC vector. Schematic diagrams of the MI-MAC vector and the multi-integrase platform are shown on top. Reporter plasmid was inserted into the φC31 attP site of the MI-MAC vector by φC31 recombinase-mediated homologous recombination in HepG2 cells. Key: Neo^r^, neomycin resistance gene; HS4, HS4 insulator; pA, polyA signal. (PPTX 45 kb)

Additional file 2: Table S1. List of Phase I, Phase II, and transporters whose gene expressions in 3D spheroid was 1.5-fold higher (*p* < 0.05) than those in feeder cells on culture day 7 (*n* = 4). (XLSX 53 kb)

Additional file 3: Figure S2. Sequential changes of albumin secretion from 3D spheroids isolated from CAG-ELuc/MI-MAC Tc mice. 3D culture was performed in the absence (open circles) or presence (filled circles) of 300 μM D-luciferin. Albumin concentration in the culture medium on the indicated culture day was measured by ELISA and divided by culture day. Error bars indicate standard deviations (*n* = 8). (PPTX 104 kb)

Additional file 4: Figure S3. Daily changes of dose-response curves of ELuc bioluminescence from compound-treated 3D spheroids shown in Figs. 4–6. The concentration response curves for every 4 days were superimposed. (PPTX 154 kb)

Additional file 5: Figure S4. Intermediate precision of luminescence measurements during experimental period. Before measurement of luminescence from 3D spheroids at each measurement point, light intensity was measured with a standard LED plate for 5 s at 37°C. (PPTX 87 kb)

## Abbreviations

3D: Three-dimensional
CAG promoter: Cytomegalovirus immediate early enhancer/chicken β-actin promoter/rabbit β-globin intron II promoter
CV: Coefficient of variation
Cy-3: Cyanine-3
DMEM: Dulbecco’s Modified Eagle’s Medium
DMSO: Dimethyl sulfoxide
ELISA: Enzyme-linked immunosorbent assay
ELuc: Emerald Luc from *Pyrearinus termitilluminans*
FBS: Fetal bovine serum
IC_50_: 50% inhibitory concentration
LDH: Lactate dehydrogenase
LED: Light emitting diode
MI-MAC vector: Multi-integrase mouse artificial chromosome vector
MTT: Methylthiazole tetrazolium
PAS: Periodic acid-Schiff
SDS: Sodium dodecyl sulfate
SV: Simian virus
Tc: Transchromosomic
WST: 2-(4-iodophenyl)-3-(4-nitrophenyl)-5-(2,4-disulfophenyl)-2*H*-tetrazolium monosodium

## References

[1] Roda A, Guardigli M, Michelini E, Mirasoli M (2009). Bioluminescence in analytical chemistry and *in vivo* imaging. Trends Anal Chem.

[2] Salipalli S, Singh PK, Borlak J (2014). Recent advances in live cell imaging of hepatoma cells. BMC Cell Biol.

[3] Natsch A, Emter R (2015). Reporter cell lines for skin sensitization testing. Arch Toxicol.

[4] Naylor LH (1999). Reporter gene technology: the future looks bright. Biochem Pharmacol.

[5] Gross S, Piwnica-Worms D (2005). Spying on cancer: molecular imaging *in vivo* with genetically encoded reporters. Cancer Cell.

[6] Nakajima Y, Ohmiya Y (2010). Bioluminescence assays: multicolor luciferase assay, secreted luciferase assay and imaging luciferase assay. Expert Opin Drug Discov.

[7] Gandelman O, Allue I, Bowers K, Cobbold P (1994). Cytoplasmic factors that affect the intensity and stability of bioluminescence from firefly luciferase in living mammalian cells. J Biolumin Chemilumin.

[8] Ignowski JM, Schaffer DV (2004). Kinetic analysis and modeling of firefly luciferase as a quantitative reporter gene in live mammalian cells. Biotechnol Bioeng.

[9] Luker KE, Luker GD (2008). Applications of bioluminescence imaging to antiviral research and therapy: multiple luciferase enzymes and quantitation. Antiviral Res.

[10] Wink S, Hiemstra S, Huppelschoten S, Danen E, Niemeijer M, Hendriks G, Vrieling H, Herpers B, van de Water B (2014). Quantitative high content imaging of cellular adaptive stress response pathways in toxicity for chemical safety assessment. Chem Res Toxicol.

[11] Rizzi N, Ramachandran B, Vantaggiato C, Ciana P, Maggi A (2014). Reporter mice for the study of long-term effects of drugs and toxic compounds. Methods Mol Biol.

[12] Wakuri S, Yamakage K, Kazuki Y, Kazuki K, Oshimura M, Aburatani S, Yasunaga M, Nakajima Y (2017). Correlation between luminescence intensity and cytotoxicity in cell-based cytotoxicity assay using luciferase. Anal Biochem.

[13] Weir LR, Schenck E, Meakin J, McClure F, Driver R, Walker S, Lynch AM (2005). Biophotonic imaging in HO-1.luc transgenic mice: real-time demonstration of gender-specific chloroform induced renal toxicity. Mutat Res.

[14] Briat A, Vassaux G (2008). A new transgenic mouse line to image chemically induced p53 activation in vivo. Cancer Sci.

[15] Chang CT, Ho TY, Lin H, Liang JA, Huang HC, Li CC, Lo HY, Wu SL, Huang YF, Hsiang CY (2012). 5-Fluorouracil induced intestinal mucositis via nuclear factor-kappaB activation by transcriptomic analysis and in vivo bioluminescence imaging. PLoS One.

[16] Godoy P, Hewitt NJ, Albrecht U, Andersen ME, Ansari N, Bhattacharya S, Bode JG, Bolleyn J, Borner C, Bottger J (2013). Recent advances in 2D and 3D in vitro systems using primary hepatocytes, alternative hepatocyte sources and non-parenchymal liver cells and their use in investigating mechanisms of hepatotoxicity, cell signaling and ADME. Arch Toxicol.

[17] Soldatow VY, Lecluyse EL, Griffith LG, Rusyn I (2013). models for liver toxicity testing. Toxicol Res.

[18] Nakajima Y, Yamazaki T, Nishii S, Noguchi T, Hoshino H, Niwa K, Viviani VR, Ohmiya Y (2010). Enhanced beetle luciferase for high-resolution bioluminescence imaging. PLoS One.

[19] Viviani VR, Silva AC, Perez GL, Santelli RV, Bechara EJ, Reinach FC (1999). Cloning and molecular characterization of the cDNA for the Brazilian larval click-beetle *Pyrearinus termitilluminans* luciferase. Photochem Photobiol.

[20] Takiguchi M, Kazuki Y, Hiramatsu K, Abe S, Iida Y, Takehara S, Nishida T, Ohbayashi T, Wakayama T, Oshimura M (2014). A novel and stable mouse artificial chromosome vector. ACS Synth Biol.

[21] Yoshimura Y, Nakamura K, Endo T, Kajitani N, Kazuki K, Kazuki Y, Kugoh H, Oshimura M, Ohbayashi T (2015). Mouse embryonic stem cells with a multi-integrase mouse artificial chromosome for transchromosomic mouse generation. Transgenic Res.

[22] Berg TO, Fengsrud M, Stromhaug PE, Berg T, Seglen PO (1998). Isolation and characterization of rat liver amphisomes. Evidence for fusion of autophagosomes with both early and late endosomes. J Biol Chem.

[23] Yasunaga M, Nakajima Y, Ohmiya Y (2014). Dual-color bioluminescence imaging assay using green- and red-emitting beetle luciferases at subcellular resolution. Anal Bioanal Chem.

[24] Yasunaga M, Murotomi K, Abe H, Yamazaki T, Nishii S, Ohbayashi T, Oshimura M, Noguchi T, Niwa K, Ohmiya Y (2015). Highly sensitive luciferase reporter assay using a potent destabilization sequence of calpain 3. J Biotechnol.

[25] Ikeda Y, Jomura T, Horiuchi U, Saeki J, Yoshimoto K, Ikeya T, Nagasaki Y (2012). Long-term survival and functional maintenance of hepatocytes by using a microfabricated cell array. Colloids Surf B: Biointerfaces.

[26] Leitao JM, da Silva JC E (2010). Firefly luciferase inhibition. J Photochem Photobiol B.

[27] Zhang H, Bai H, Jiang T, Ma Z, Cheng Y, Zhou Y, Du L, Li M (2016). Quenching the firefly bioluminescence by various ions. Photochem Photobiol Sci.

[28] Thorne N, Shen M, Lea WA, Simeonov A, Lovell S, Auld DS, Inglese J (2012). Firefly luciferase in chemical biology: a compendium of inhibitors, mechanistic evaluation of chemotypes, and suggested use as a reporter. Chem Biol.

[29] Patrick PS, Lyons SK, Rodrigues TB, Brindle KM (2014). Oatp1 enhances bioluminescence by acting as a plasma membrane transporter for D-luciferin. Mol Imaging Biol.

[30] Oshimura M, Uno N, Kazuki Y, Katoh M, Inoue T (2015). A pathway from chromosome transfer to engineering resulting in human and mouse artificial chromosomes for a variety of applications to bio-medical challenges. Chromosome Res.

[31] Yamaguchi S, Kazuki Y, Nakayama Y, Nanba E, Oshimura M, Ohbayashi T (2011). A method for producing transgenic cells using a multi-integrase system on a human artificial chromosome vector. PLoS One.

