# Supplementary material for: Continuous long-term cytotoxicity monitoring in 3D spheroids of beetle luciferase-expressing hepatocytes by nondestructive bioluminescence measurement
Source: BMC Biotechnol. 2017 Jun 20;17:54. doi: 10.1186/s12896-017-0374-1 (PMC5480146; doi:10.1186/s12896-017-0374-1)
Supplement: Supplementary file 1 — Schematic diagram of site-specific insertion of reporter plasmid carrying ELuc into MI-MAC vector. Schematic diagrams of the MI-MAC vector and the multi-integrase platform are shown on top. Reporter plasmid was inserted into the φC31 attP site of the MI-MAC vector by φC31 recombinase-mediated homologous recombination in HepG2 cells. Key: Neor, neomycin resistance gene; HS4, HS4 insulator; pA, polyA signal. (PPTX 45 kb) [file 12896_2017_374_MOESM1_ESM.pptx]

## Slide 1
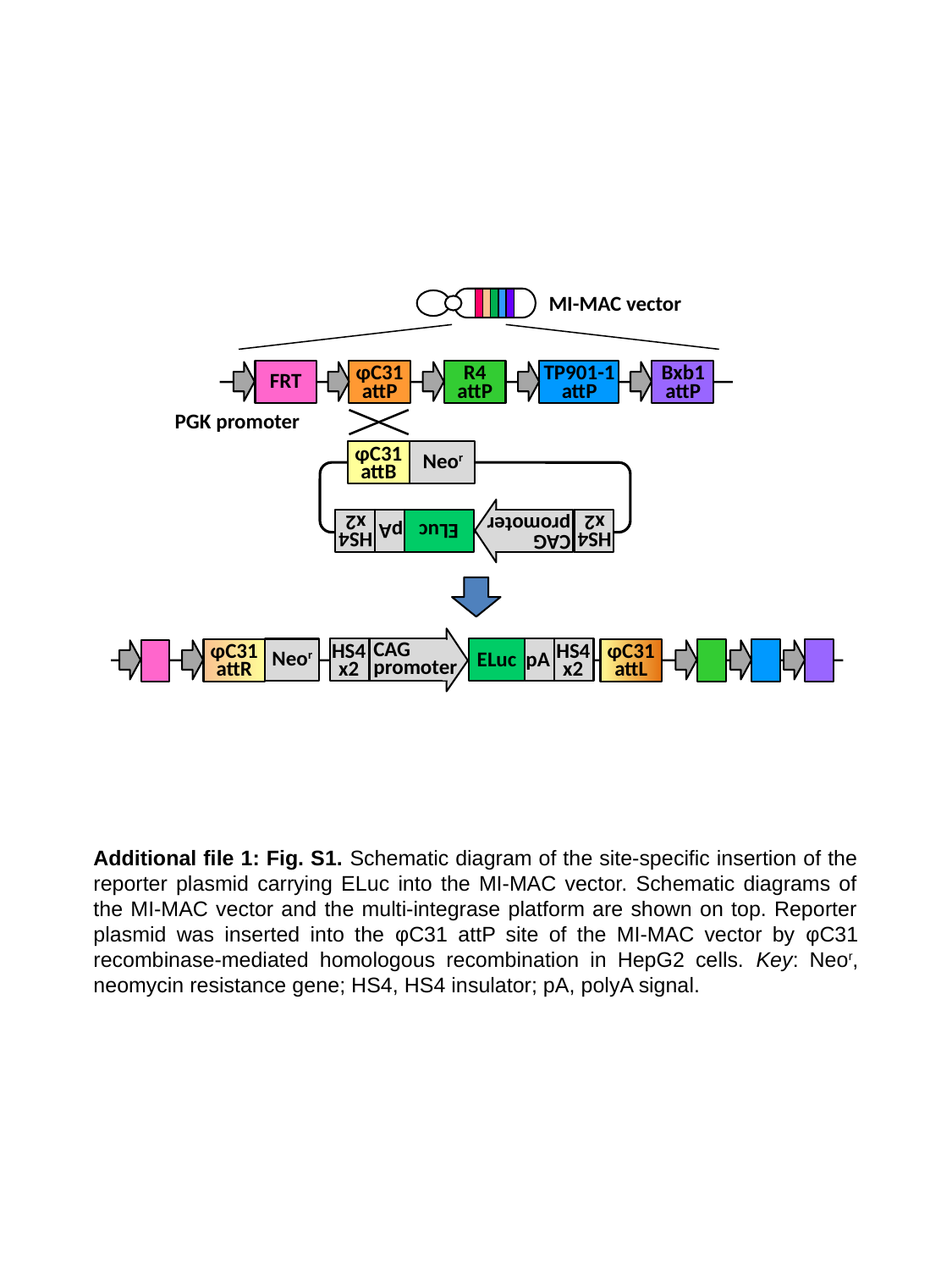

MI-MAC vector
φC31
attP
R4
attP
TP901-1
attP
Bxb1
attP
FRT
PGK promoter
φC31
attB
Neor
HS4
x2
HS4
x2
CAG
promoter
ELuc
pA
CAG
promoter
HS4
x2
HS4
x2
φC31
attR
φC31
attL
Neor
ELuc
pA
Additional file 1: Fig. S1. Schematic diagram of the site-specific insertion of the reporter plasmid carrying ELuc into the MI-MAC vector. Schematic diagrams of the MI-MAC vector and the multi-integrase platform are shown on top. Reporter plasmid was inserted into the φC31 attP site of the MI-MAC vector by φC31 recombinase-mediated homologous recombination in HepG2 cells. Key: Neor, neomycin resistance gene; HS4, HS4 insulator; pA, polyA signal.
