# Supplementary material for: Continuous long-term cytotoxicity monitoring in 3D spheroids of beetle luciferase-expressing hepatocytes by nondestructive bioluminescence measurement
Source: BMC Biotechnol. 2017 Jun 20;17:54. doi: 10.1186/s12896-017-0374-1 (PMC5480146; doi:10.1186/s12896-017-0374-1)
Supplement: Supplementary file 3 — Sequential changes of albumin secretion from 3D spheroids isolated from CAG-ELuc/MI-MAC Tc mice. 3D culture was performed in the absence (open circles) or presence (filled circles) of 300 μM D-luciferin. Albumin concentration in the culture medium on the indicated culture day was measured by ELISA and divided by culture day. Error bars indicate standard deviations (n = 8). (PPTX 104 kb) [file 12896_2017_374_MOESM3_ESM.pptx]

## Slide 1
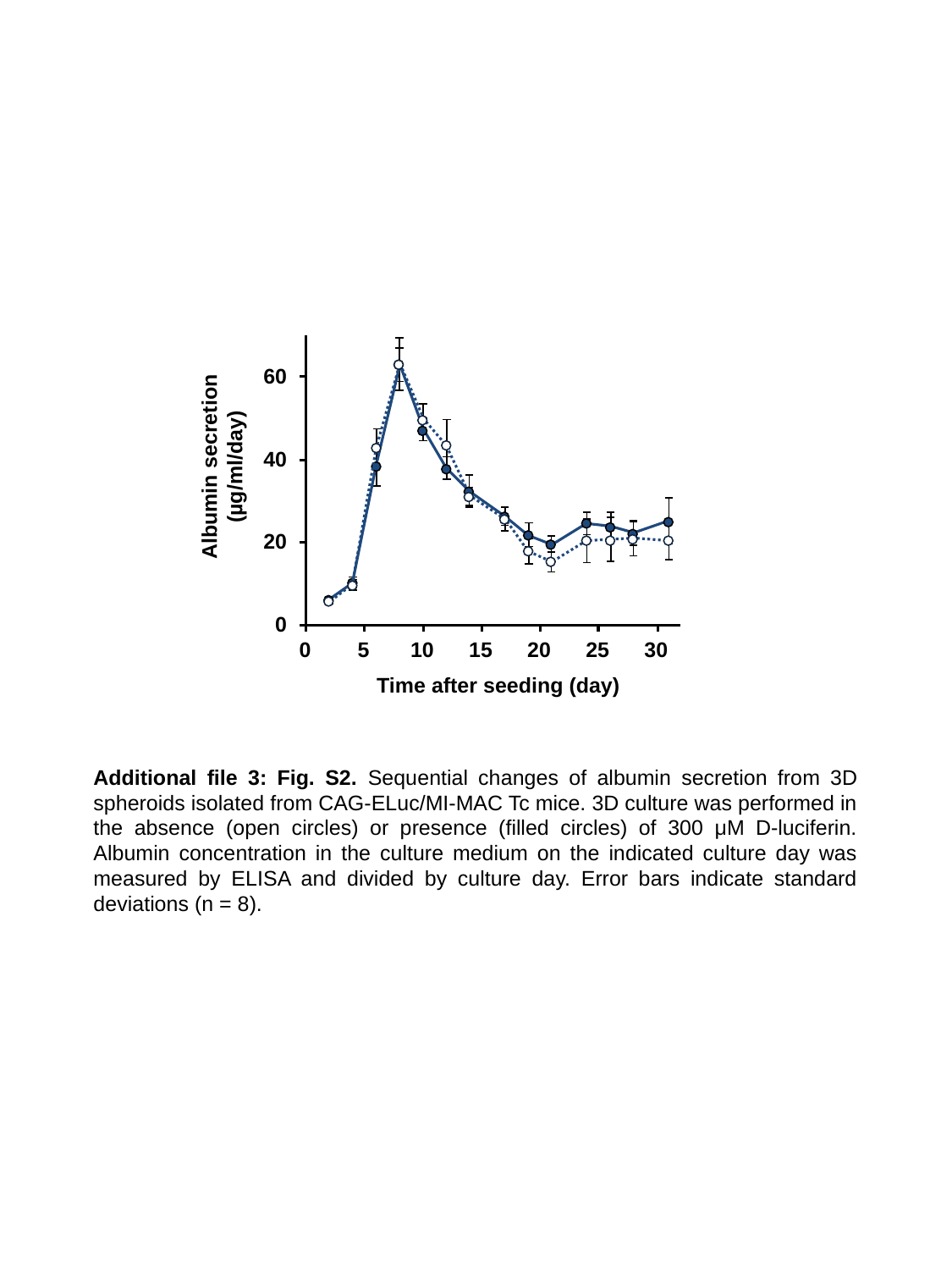

Albumin secretion
(µg/ml/day)
Time after seeding (day)
Additional file 3: Fig. S2. Sequential changes of albumin secretion from 3D spheroids isolated from CAG-ELuc/MI-MAC Tc mice. 3D culture was performed in the absence (open circles) or presence (filled circles) of 300 μM D-luciferin. Albumin concentration in the culture medium on the indicated culture day was measured by ELISA and divided by culture day. Error bars indicate standard deviations (n = 8).
