# Supplementary material for: Continuous long-term cytotoxicity monitoring in 3D spheroids of beetle luciferase-expressing hepatocytes by nondestructive bioluminescence measurement
Source: BMC Biotechnol. 2017 Jun 20;17:54. doi: 10.1186/s12896-017-0374-1 (PMC5480146; doi:10.1186/s12896-017-0374-1)
Supplement: Supplementary file 4 — Daily changes of dose-response curves of ELuc bioluminescence from compound-treated 3D spheroids shown in Figs. 4–6. The concentration response curves for every 4 days were superimposed. (PPTX 154 kb) [file 12896_2017_374_MOESM4_ESM.pptx]

## Slide 1
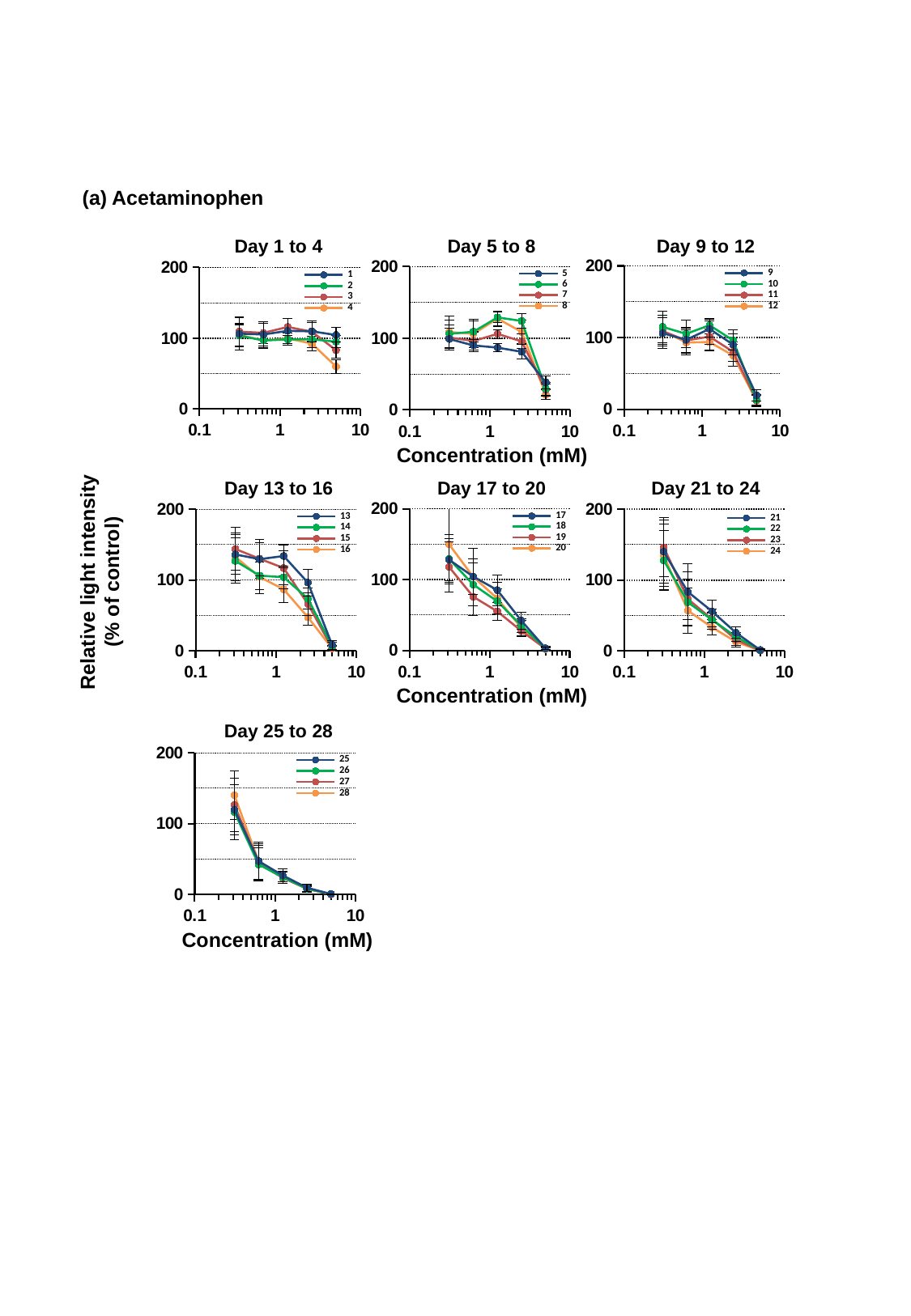

(a) Acetaminophen
Day 1 to 4
Day 5 to 8
Day 9 to 12
### Chart
| Category | | | | |
|---|---|---|---|---|
### Chart
| Category | | | | |
|---|---|---|---|---|
### Chart
| Category | | | | |
|---|---|---|---|---|Concentration (mM)
Relative light intensity
(% of control)
Day 13 to 16
Day 17 to 20
Day 21 to 24
### Chart
| Category | | | | |
|---|---|---|---|---|
### Chart
| Category | | | | |
|---|---|---|---|---|
### Chart
| Category | | | | |
|---|---|---|---|---|Concentration (mM)
Day 25 to 28
### Chart
| Category | | | | |
|---|---|---|---|---|Concentration (mM)

## Slide 2
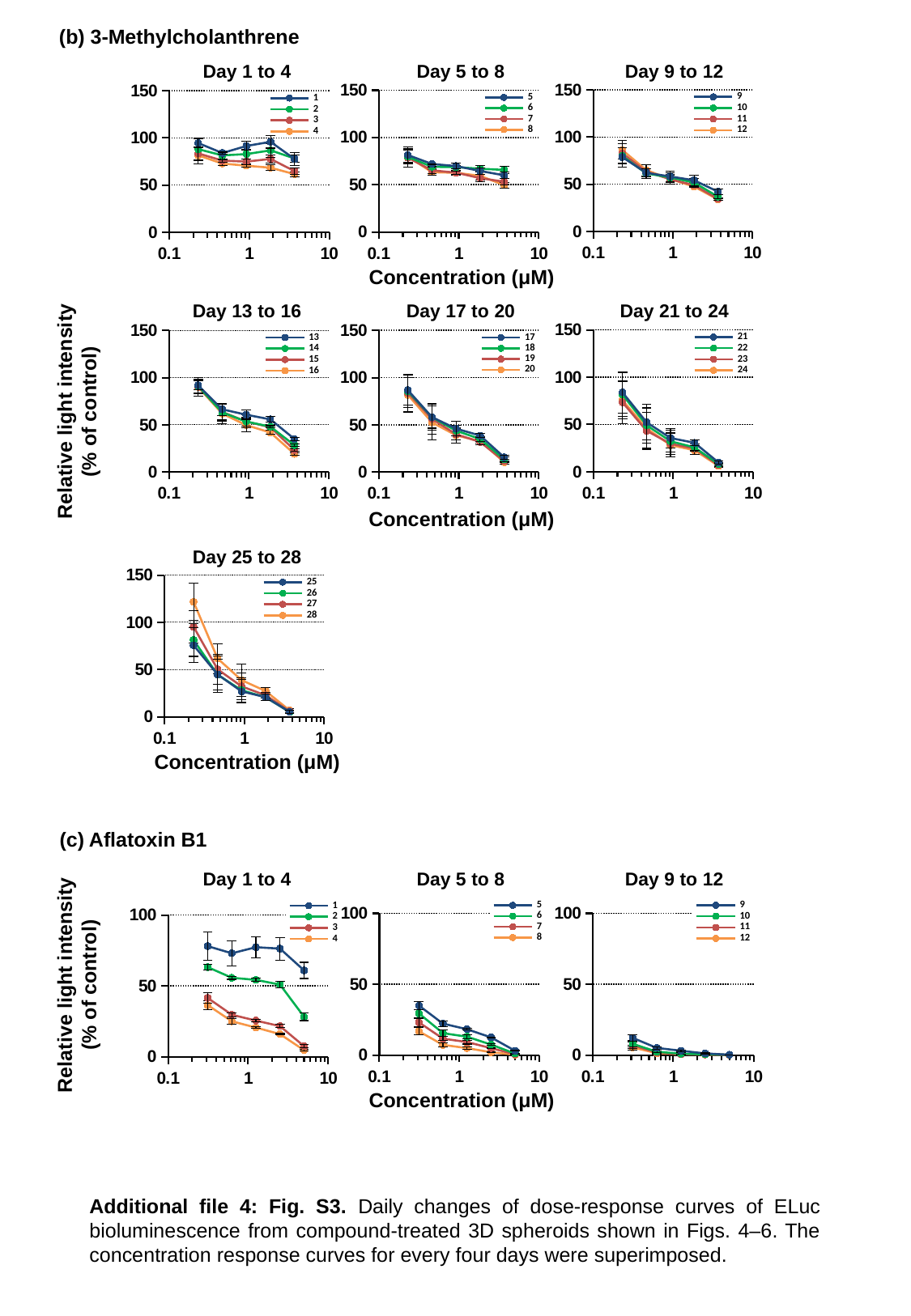

(b) 3-Methylcholanthrene
Day 1 to 4
Day 5 to 8
Day 9 to 12
### Chart
| Category | | | | |
|---|---|---|---|---|
### Chart
| Category | | | | |
|---|---|---|---|---|
### Chart
| Category | | | | |
|---|---|---|---|---|Concentration (μM)
Day 13 to 16
Day 17 to 20
Day 21 to 24
Relative light intensity
(% of control)
### Chart
| Category | | | | |
|---|---|---|---|---|
### Chart
| Category | | | | |
|---|---|---|---|---|
### Chart
| Category | | | | |
|---|---|---|---|---|Concentration (μM)
Day 25 to 28
### Chart
| Category | | | | |
|---|---|---|---|---|Concentration (μM)
(c) Aflatoxin B1
Day 1 to 4
Day 5 to 8
Day 9 to 12
Relative light intensity
(% of control)
### Chart
| Category | | | | |
|---|---|---|---|---|
### Chart
| Category | | | | |
|---|---|---|---|---|
### Chart
| Category | | | | |
|---|---|---|---|---|Concentration (μM)
Additional file 4: Fig. S3. Daily changes of dose-response curves of ELuc bioluminescence from compound-treated 3D spheroids shown in Figs. 4‒6. The concentration response curves for every four days were superimposed.
