# Supplementary material for: Continuous long-term cytotoxicity monitoring in 3D spheroids of beetle luciferase-expressing hepatocytes by nondestructive bioluminescence measurement
Source: BMC Biotechnol. 2017 Jun 20;17:54. doi: 10.1186/s12896-017-0374-1 (PMC5480146; doi:10.1186/s12896-017-0374-1)
Supplement: Supplementary file 5 — Intermediate precision of luminescence measurements during experimental period. Before measurement of luminescence from 3D spheroids at each measurement point, light intensity was measured with a standard LED plate for 5 s at 37°C. (PPTX 87 kb) [file 12896_2017_374_MOESM5_ESM.pptx]

## Slide 1
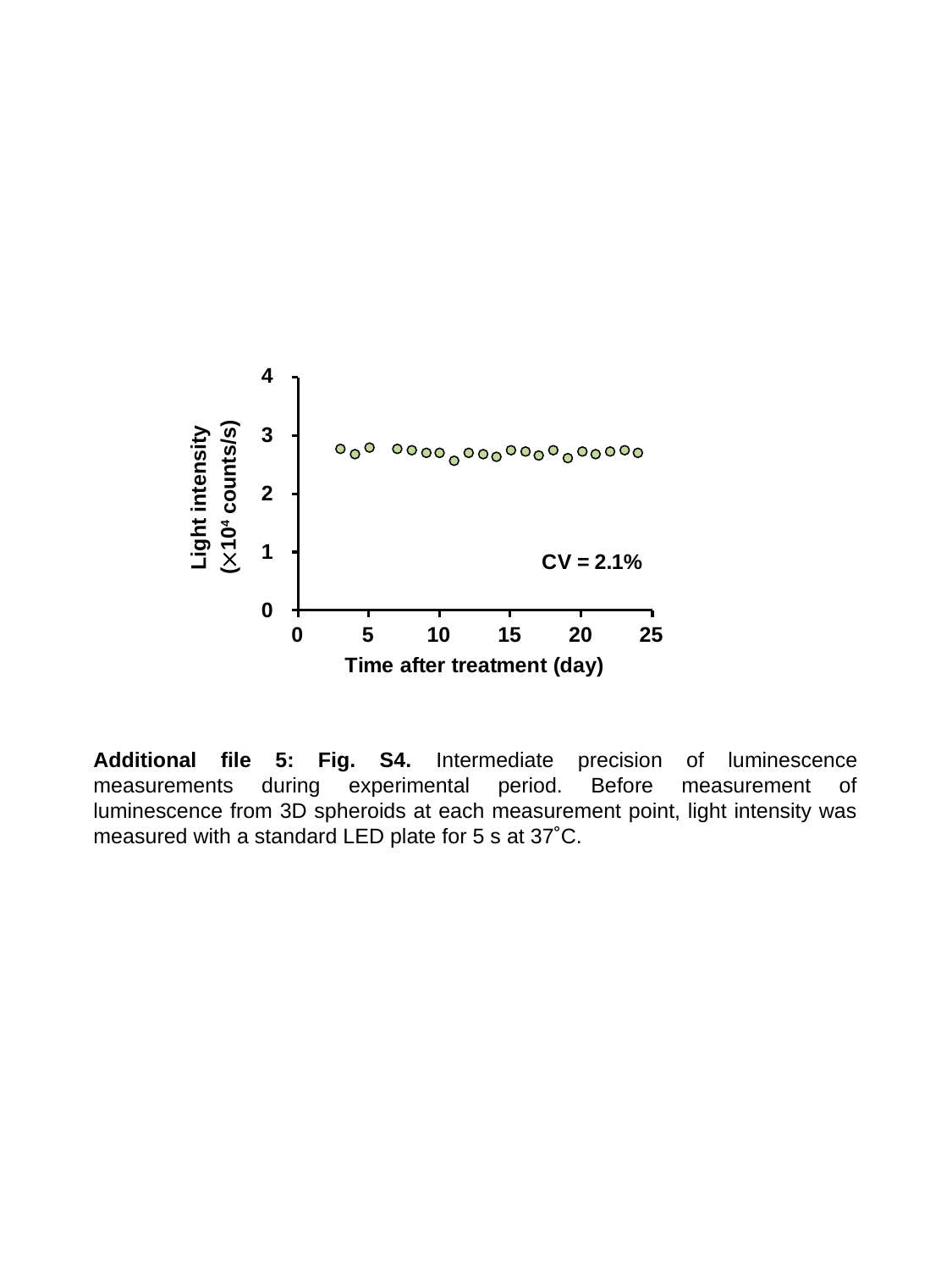

Light intensity
(×104 counts/s)
Additional file 5: Fig. S4. Intermediate precision of luminescence measurements during experimental period. Before measurement of luminescence from 3D spheroids at each measurement point, light intensity was measured with a standard LED plate for 5 s at 37˚C.
